# Benefit of prompt initiation of single-inhaler fluticasone furoate, umeclidinium, and vilanterol (FF/UMEC/VI) in patients with COPD in England following an exacerbation: a retrospective cohort study

**DOI:** 10.1186/s12931-023-02523-1

**Published:** 2023-09-25

**Authors:** Afisi S. Ismaila, Kieran J. Rothnie, Robert P. Wood, Victoria L. Banks, Lucinda J. Camidge, Alexandrosz Czira, Chris Compton, Raj Sharma, Shannon N. Millard, Olivia Massey, David M. G. Halpin

**Affiliations:** 1 Value Evidence and Outcomes, R&D Global Medical, GSK, 1250 South Collegeville Road, Collegeville, PA USA; 2 Department of Health Research Methods, Evidence and Impact, McMaster University, Hamilton, ON Canada; 3 Value Evidence and Outcomes, R&D Global Medical, GSK, London, UK; 4 Real-World Evidence, Adelphi Real World, Bollington, Cheshire UK; 5 Integrated Evidence Generation (Women’s Health Care), Bayer PLC, Reading, UK; 6 Global Medical, GSK, London, UK; 7 P1vital Limited, Wallingford, Oxfordshire UK; 8 College of Medicine and Health, University of Exeter Medical School, University of Exeter, Exeter, UK

**Keywords:** Chronic obstructive pulmonary disease, Exacerbation, FF/UMEC/VI, Healthcare cost, SITT

## Abstract

**Background:**

Triple therapy is recommended for patients with chronic obstructive pulmonary disease (COPD) who remain symptomatic despite dual therapy. The optimal timing of triple therapy following an exacerbation of COPD is unknown. The outcomes of prompt (≤ 30 days) vs. delayed (31–180 days) initiation of single-inhaler triple therapy with fluticasone furoate, umeclidinium, and vilanterol (FF/UMEC/VI) following an exacerbation of COPD were examined.

**Methods:**

This was a retrospective cohort study of linked English primary (Clinical Practice Research Datalink) and secondary (Hospital Episode Statistics) care data. Patients aged ≥ 35 years with COPD were indexed on the first and/or earliest date of exacerbation between November 15, 2017 and March 31, 2019 with subsequent FF/UMEC/VI initiation within 180 days. Patients were required to be continuously registered with a general practitioner for ≥ 12 months prior to and following index. Subsequent exacerbations, direct medical costs, and hospital readmissions were compared between prompt and delayed initiators. Inverse probability of treatment weighting was used to adjust for measured confounders between cohorts.

**Results:**

Overall, 1599 patients were included (prompt: 393, delayed: 1206). After weighting, prompt initiators had numerically lower moderate/severe exacerbations compared with delayed initiators (rate ratio: 0.87, 95% confidence interval [CI]: 0.76–1.01, p = 0.0587). Both all-cause and COPD-related 30-day hospital readmissions were significantly lower among patients with prompt initiation compared with delayed initiators (all-cause: 23.6% vs. 34.6%, odds ratio [95% CI]: 0.58 [0.36–0.95], p = 0.0293; COPD-related: 20.3% vs. 30.6%, odds ratio [95% CI]: 0.58 [0.35–0.96], p = 0.0347). Prompt initiators also had numerically lower all-cause total costs and significantly lower COPD-related costs per-person-per year compared with delayed initiators (COPD-related: £742 vs. £801, p = 0.0016).

**Conclusion:**

Prompt initiation of FF/UMEC/VI following a moderate/severe exacerbation was associated with fewer subsequent exacerbations, fewer hospital readmissions, and lower COPD-related medical costs compared with delayed initiation.

**Supplementary Information:**

The online version contains supplementary material available at 10.1186/s12931-023-02523-1.

## Plain language summary

Triple therapy with an inhaled corticosteroid (ICS), a long-acting muscarinic antagonist (LAMA), and a long-acting β2-agonist (LABA) is recommended for patients with chronic obstructive pulmonary disease (COPD) who still experience symptoms while taking dual therapy (LABA/LAMA or ICS/LABA). Triple therapy can be taken using single or multiple inhalers. The best time to start triple therapy for patients who may benefit from it following a short-term worsening (flare-up) of their COPD symptoms is unknown. This study assesses the effect of starting treatment with triple therapy sooner compared with later in patients with COPD.

Patients who experienced a flare-up of their COPD symptoms were split into two groups – those who started taking triple therapy (via a single inhaler) within 30 days of their symptom flare-up and those who started taking triple therapy 31–180 days following their symptom flare-up. Over the 12 months following the initial flare-up, patients who started triple therapy earlier (within 30 days) had fewer subsequent symptom flare-ups, fewer hospital admissions, and lower healthcare costs compared with patients who started triple therapy later (31–180 days). These findings suggest that doctors should consider prescribing triple therapy (via a single inhaler) to their patients with COPD straight away if they experience a flare-up of their symptoms.

**Supplementary Information:**

The online version contains supplementary material available at 10.1186/s12931-023-02523-1.

## Introduction

Escalation to triple therapy with an inhaled corticosteroid (ICS), a long-acting muscarinic antagonist (LAMA), and a long-acting β_2_-agonist (LABA) is recommended for patients with chronic obstructive pulmonary disease (COPD) who continue to experience exacerbations despite dual therapy with LAMA/LABA [1]. Traditionally, triple therapy has required the use of two or three separate inhalers (multiple-inhaler triple therapy; MITT); however, more recently, single-inhaler triple therapies (SITTs) have been developed. SITTs can help facilitate greater treatment persistence and adherence by reducing the burden of the mode of administration. MITT has been shown to be associated with an increased risk of treatment discontinuation and reduced adherence compared with single-inhaler use [2–4].

Once-daily SITT with fluticasone furoate/umeclidinium/vilanterol (FF/UMEC/VI) was approved for the long-term maintenance of moderate-to-severe COPD in adult patients who are not adequately treated by a combination of ICS/LABA or LABA/LAMA in Europe in November 2017 [5]. Previous clinical trials have demonstrated that patients with COPD initiated on FF/UMEC/VI experience reduced rates of exacerbations (moderate or severe) and lower rates of COPD-related hospitalizations compared with patients receiving dual therapy with ICS/LABA or LABA/LAMA [6].

Although previous studies have demonstrated that prompt vs. delayed initiation of MITT following an exacerbation of COPD reduces subsequent exacerbations and medical costs [7, 8], there is limited real-world evidence of the consequences of delaying initiation of SITT when indicated. A recent retrospective study in the US reported that prompt initiation (within 30 days of an exacerbation) of SITT with FF/UMEC/VI following a moderate or severe COPD exacerbation was associated with significantly fewer subsequent exacerbations and lower healthcare costs compared with delayed initiation of FF/UMEC/VI (within 31–180 days of an exacerbation) [9]. However, these findings may be specific to this healthcare system, and the effects of delayed initiation have not been assessed for patients in England.

The aim of this study was to assess the outcomes of prompt (0–30 days following an exacerbation) vs. delayed (31–180 days following an exacerbation) initiation of SITT with FF/UMEC/VI among a general practice cohort of patients with COPD in England.

## Material and methods

### Study design and data source

This was a new user, retrospective, weighted cohort study of English patients with COPD using UK primary care electronic health records (Clinical Practice Research Datalink [CPRD] Aurum) and linked secondary care data (Hospital Episode Statistics [HES] Admitted Patient Care and Accident and Emergency [A&E] datasets).

CPRD Aurum is a longitudinal, anonymized, electronic health record database of primary care interactions for all patients registered with a participating general practitioner (GP) practice in the UK [10]. Data captured include demographic information (age, sex, weight); records of clinical events (medical diagnoses); immunization records; diagnostic testing; lifestyle information (e.g., smoking status and alcohol status); and all other types of care administered as part of routine GP practice. Linkage to HES is possible for a subset of patients registered at GP practices throughout England. HES is a database containing details of all secondary episodes of care (e.g., inpatient admissions, day cases, outpatient appointments, and A&E attendances).

**Fig. 1:**
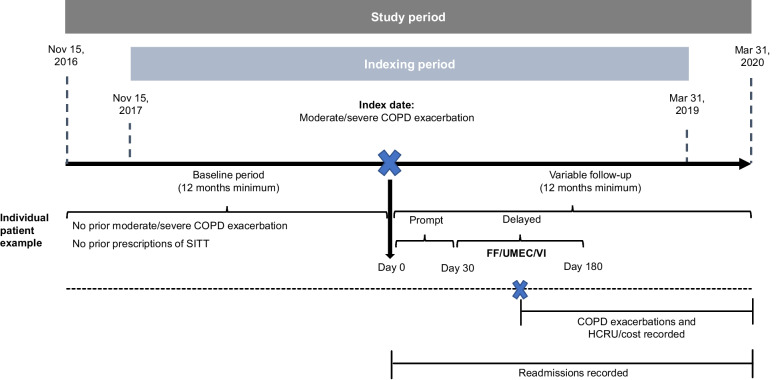
Study design. *COPD* chronic obstructive pulmonary disease, *SITT* single-inhaler triple therapy, *FF/UMEC/VI* fluticasone furoate/umeclidinium/vilanterol, *HCRU* healthcare resource utilization

Patients were indexed on the first and/or earliest date of exacerbation of COPD (moderate or severe) between November 15, 2017 (approval date of FF/UMEC/VI in Europe) and March 31, 2019 (Fig. 1).

COPD exacerbations were identified from CPRD and HES based on a validated algorithm [11, 12]. Exacerbations resulting in hospitalization (i.e., recorded in HES) were considered severe, while exacerbations managed only in primary care (i.e., only recorded in CPRD) were considered moderate. The baseline period was defined as the 12 months prior to index; the minimum follow-up (from and including the index date) was 12 months. The follow-up period spanned from the index date until either the end of the study period on March 31, 2020, the end of data availability (the date that the patient left the GP practice or the last data collection date of the practice), or patient death, whichever was earliest. Of note, the entire study period was prior to the emergence of COVID-19 in the UK; COPD patient management was found to differ during the pandemic and the change in healthcare service was not under study [13].

Two mutually exclusive cohorts were defined. Patients were classified as prompt initiators if FF/UMEC/VI therapy was initiated within 0‒30 days of the index date, and delayed initiators if FF/UMEC/VI therapy was initiated within 31‒180 days of the index date. A pragmatic approach was adopted, whereby patients who discontinued FF/UMEC/VI within the follow-up period were still observed and continued to be assessed until the end of follow-up. This pragmatic approach allowed us to assess the research question of the impact of a policy of prompt initiation of FF/UMEC/VI. As an additional analysis, initiation of FF/UMEC/VI within 0–14 days of the index exacerbation was considered prompt, and initiation of FF/UMEC/VI within 15–180 days of the index exacerbation was considered delayed. Inverse probability of treatment weighting (IPTW) based on propensity scores (PSs) was used to adjust for measured confounders between cohorts. Different PSs were used for the assessment of each outcome. Covariates included in the PS model are outlined in Additional file 1: Table S1.

### Study population

Patients were required to have at least one diagnosis of COPD at ≥ 35 years of age (in line with guidance from the National Institute for Health and Care Excellence [14]); ≥ 1 moderate or severe exacerbation within the indexing period (November 15, 2017 to March 31, 2019); ≥ 1 prescription for FF/UMEC/VI on or within 180 days of the index date; most recent smoking status prior to index of “current smoker” or “former smoker”; records linked to HES; and be continuously registered with a GP practice for ≥ 12 months prior to the index date and ≥ 12 months following index.

Patients were excluded if they had ≥ 1 exacerbation (moderate or severe) during the baseline period; ≥ 1 prescription for FF/UMEC/VI or SITT beclomethasone dipropionate/formoterol fumarate/glycopyrronium bromide (BDP/FOR/GLY) prior to the index date; ≥ 1 prescription for BDP/FOR/GLY between the index date and FF/UMEC/VI initiation; or ≥ 1 diagnostic code for any medical condition incompatible with a COPD diagnosis at any time in their medical history prior to indexing.

### Study outcomes

#### Primary objective

The primary objective was to compare the rate of subsequent moderate/severe exacerbations among prompt vs. delayed initiators of FF/UMEC/VI. Exacerbations were identified using a validated algorithm [11, 12]. For further details please see Additional file 1. Unweighted and weighted rates of exacerbations (frequency of events per person-year) were calculated as the number of events observed divided by person-years of observation. Rates were reported as overall (moderate and severe exacerbations) and separately by severity, and were compared between cohorts using IPTW-weighted rate ratios (RRs), 95% confidence intervals (CIs), and p-values obtained from negative binomial regression. The rate of subsequent exacerbations, when stratified by severity of index exacerbation, was also calculated. An additional post-hoc analysis, censoring patients at initiation of FF/UMEC/VI for the delayed cohort, was conducted to investigate how much of the impact of prompt initiation was due to the efficacy of FF/UMEC/VI vs. therapy at the time of exacerbation rather than due to prompt initiation.

#### Secondary objectives

The secondary objectives were to compare time-to-first subsequent exacerbation, hospital readmissions, healthcare resource utilization (HCRU), and direct medical costs among prompt vs. delayed initiators of FF/UMEC/VI.

##### Time-to-first subsequent exacerbation

Exacerbations were identified as per the algorithm and definitions used for the primary objective. Time-to-first exacerbation was measured from initiation of FF/UMEC/VI and compared between cohorts. Time-to-first exacerbation was assessed using Kaplan–Meier (KM) survival analysis. Unweighted and weighted KM survival curves were produced, and time-to-first exacerbation was compared between cohorts using IPTW-weighted hazard ratios (HRs), 95% CIs, and p-values from Cox proportional hazards regression.

##### Hospital readmissions

The absolute proportion of hospital readmissions for prompt and delayed initiators was derived in the 30, 60, and 90 days following the index date for the subset of patients who were indexed on a severe exacerbation (i.e., requiring hospital admission), based on the presence of an inpatient date of readmission. Readmissions were defined as COPD-related or all-cause based on the presence of a primary or secondary diagnosis of COPD (using International Classification of Disease, 10^th^ Revision, codes). Time-to-first hospital readmission was also measured from the index date and compared between cohorts using KM survival analysis. The unweighted and weighted proportion of hospital readmissions was evaluated. Comparisons between cohorts were performed using HRs, 95% CIs, and p-values from IPTW-weighted univariable logistic regression.

##### HCRU and costs

All-cause and COPD-related HCRU and direct medical costs following initiation of FF/UMEC/VI were calculated and compared between the prompt and delayed cohorts. HCRU was reported as rates (frequency of events per person-year) and costs were reported as per-person-per-year to account for the variable follow-up across patients. HCRU was compared between cohorts using weighted RRs, 95% CIs, and p-values obtained from negative binomial regression. Costs were derived using the most recent source document at the time of analysis (up to 2020 in line with the study period end). For prescriptions written in primary care, direct healthcare costs were calculated via the application of cost-per-unit from the April 2019–March 2020 NHS Drug Tariff [15]. For primary care consultations and interactions in a hospital setting (i.e., inpatient admissions, outpatient appointments, and A&E visits), direct healthcare costs were calculated via application of unit costs from the 2020 Personal Social Service Resource Unit [16] and via application of national tariffs to healthcare resource groups, respectively [17, 18]. Costs were compared between cohorts using weighted exponentiated coefficients, 95% CIs, and p-values obtained from generalized linear model with log link and gamma distribution.

#### Exploratory objective

The exploratory objective was to evaluate the association between rate of subsequent exacerbations of COPD following index exacerbation and time-to-initiation of FF/UMEC/VI as a continuous variable. The impact of time-to-initiation of FF/UMEC/VI on the rate of subsequent exacerbations was evaluated using unweighted RRs, 95% CIs, and p-values obtained from negative binomial regression, adjusting for covariates.

## Results

### Baseline demographics

**Fig. 2:**
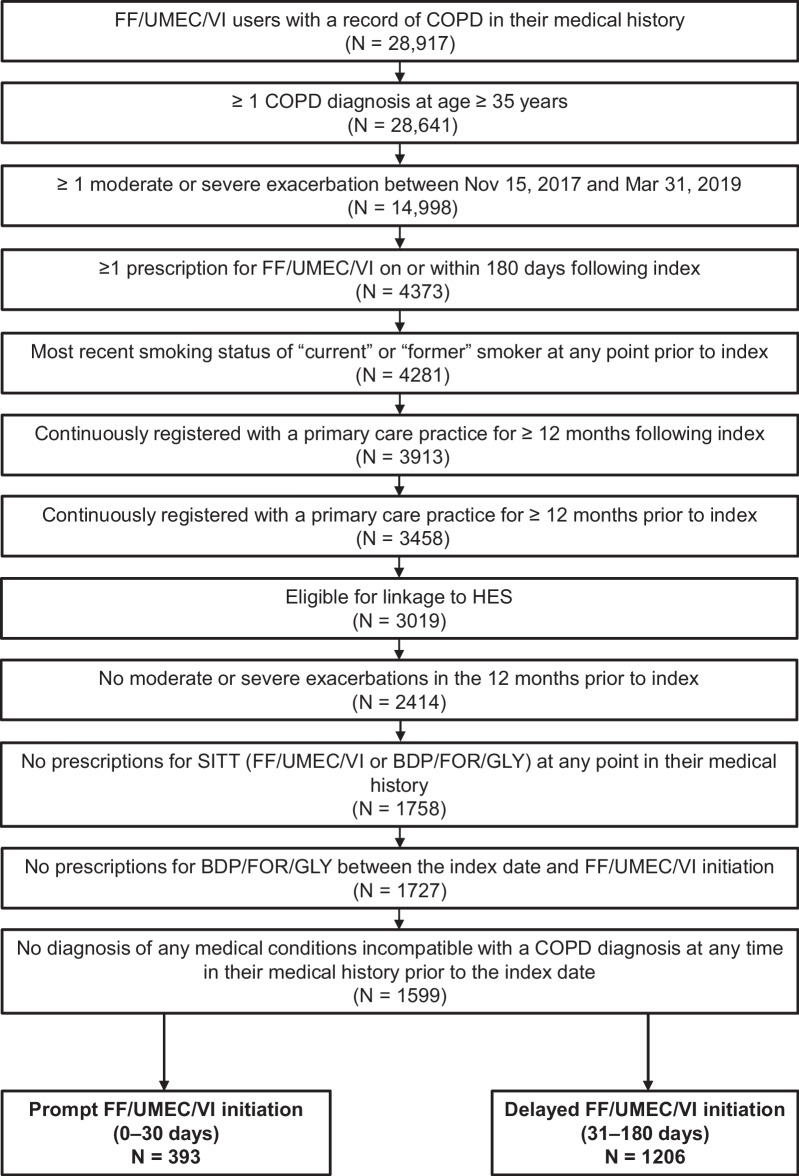
Patient attrition. *FF/UMEC/VI* fluticasone furoate/umeclidinium/vilanterol, *COPD* chronic obstructive pulmonary disease, *HES* Hospital Episode Statistics, *SITT* single-inhaler triple therapy, *BDP/FOR/GLY* beclomethasone dipropionate/formoterol fumarate/glycopyrronium bromide

A total of 1599 patients met the eligibility criteria and were included in the study (Fig. 2).

Overall, 393 patients (24.6%) had prompt (≤ 30 days from index) initiation of FF/UMEC/VI and 1206 patients (75.4%) had delayed (31–180 days from index) initiation. Of the 393 patients in the prompt cohort, 87 patients (22.1%) initiated FF/UMEC/VI within the first 3 days following the index exacerbation (Additional file 1: Fig. S1). In the additional analysis (when initiation of FF/UMEC/VI within 0–14 days of the index exacerbation was considered prompt), 223 patients (13.9%) were categorized as prompt initiators and 1376 patients (86.1%) were categorized as delayed initiators.

Baseline demographics were similar between the prompt and the delayed cohorts, including forced expiratory volume in 1 s percent predicted and distribution of Medical Research Council Dyspnea Scale score (Table 1).

**Table 1:** Baseline demographics of patients overall, and stratified by prompt or delayed initiation of FF/UMEC/VI

	Total (N = 1599)	Prompt (0–30 days) (N = 393)	Delayed (31–180 days) (N = 1206)
Age at index (years)			
Mean (SD)	69.8 (10.3)	69.5 (9.8)	69.9 (10.5)
Sex, n (%)			
Male	847 (53.0)	218 (55.5)	629 (52.2)
Ethnicity, n (%)			
White	1520 (95.1)	376 (95.7)	1144 (94.9)
Other	22 (1.4)	5 (1.3)	17 (1.4)
Unknown	57 (3.6)	12 (3.1)	45 (3.7)
Smoking status, n (%)			
Current smoker	811 (50.7)	216 (55.0)	595 (49.3)
Former smoker	788 (49.3)	177 (45.0)	611 (50.7)
BMI (kg/m^2^)	n = 1429	n = 354	n = 1075
Mean (SD)	27.3 (6.3)	27.2 (6.4)	27.40 (6.3)
FEV_1_/FVC ratio	n = 973	n = 251	n = 722
Mean (SD)	56.3 (14.5)	55.1 (13.7)	56.72 (14.8)
FEV_1_% predicted	n = 1289	n = 326	n = 963
Mean (SD)	55.7 (19.5)	53.5 (18.7)	56.41 (19.7)
MRC Dyspnea Scale score, n (%)			
Grade 1	114 (7.1)	26 (6.6)	88 (7.3)
Grade 2	447 (28.0)	110 (28.0)	337 (27.9)
Grade 3	491 (30.7)	118 (30.0)	373 (30.9)
Grade 4	322 (20.1)	97 (24.7)	225 (18.7)
Grade 5	66 (4.1)	15 (3.8)	51 (4.2)
Unknown	159 (9.9)	27 (6.9)	132 (11.0)
Comorbidities*, n (%)			
Depression	738 (46.2)	190 (48.4)	548 (45.4)
Rheumatoid/osteo arthritis	612 (38.3)	136 (34.6)	476 (39.5)
Anxiety	505 (31.6)	115 (29.3)	390 (32.3)
Gastroesophageal reflux disease	450 (28.1)	114 (29.0)	336 (27.9)
Diabetes	359 (22.5)	88 (22.4)	271 (22.5)
Stroke	194 (12.1)	42 (10.7)	152 (12.6)
Acute myocardial infarction	186 (11.6)	45 (11.5)	141 (11.7)
Congestive heart failure	172 (10.8)	52 (13.2)	120 (10.0)
Dementia/cognitive impairment	170 (10.6)	39 (9.9)	131 (10.9)
Bronchiectasis	113 (7.1)	30 (7.6)	83 (6.9)
Lung cancer	23 (1.4)	5 (1.3)	18 (1.5)

*FF/UMEC/VI* fluticasone furoate/umeclidinium/vilanterol, *SD* standard deviation, *BMI* body mass index, *FEV*_*1*_ forced expiratory volume in 1 s, *FVC* forced vital capacity, *MRC* Medical Research Council, *SNOMED-CT* Systematized Nomenclature of Medicine Clinical Terms, *ICD-10* International Classification of Disease, 10^th^ Revision

*The presence of specific comorbidities prior to the index date was reported for all patients based on the presence of diagnosis codes (SNOMED-CT and ICD-10) in the patient’s entire medical history

The proportion of current smokers was slightly higher in the prompt cohort compared with the delayed cohort (55.0% vs. 49.3%). The mean number of medication classes/treatment strategies received within the 12-month baseline period was approximately three for both cohorts (Table 2).

**Table 2:** Treatment patterns at baseline for all patients and stratified by prompt or delayed FF/UMEC/VI initiation

	Total (N = 1599)	Prompt (0–30 days) (N = 393)	Delayed (31–180 days) (N = 1206)
Number of respiratory therapy classes at baseline			
Mean (SD)	3.0 (1.22)	3.1 (1.14)	3.0 (1.24)
Class of respiratory therapy at baseline*, n (%)			
SABA	1426 (89.2)	358 (91.1)	1068 (88.6)
MITT	1005 (62.9)	259 (65.9)	746 (61.9)
ICS/LABA	952 (59.5)	233 (59.3)	719 (59.6)
LAMA	814 (50.9)	197 (50.1)	617 (51.2)
LABA/LAMA	313 (19.6)	101 (25.7)	212 (17.6)
Methylxanthine	98 (6.1)	27 (6.9)	71 (5.9)
ICS/SABA	73 (4.6)	14 (3.6)	59 (4.9)
Inhaled therapy regimen immediately prior to index^†^, n (%)			
MITT	731 (45.7)	168 (42.8)	563 (46.7)
ICS, LABA, or ICS/LABA	352 (22.0)	75 (19.1)	277 (23.0)
LABA/LAMA	257 (16.1)	91 (23.2)	166 (13.8)
LAMA	181 (11.3)	49 (12.5)	132 (11.0)
None of the above regimens	78 (4.9)	10 (2.5)	68 (5.6)

*FF/UMEC/VI* fluticasone furoate/umeclidinium/vilanterol, *SD* standard deviation, *SABA* short-acting β_2_-agonist, *MITT* multiple-inhaler triple therapy, *ICS* inhaled corticosteroid, *LABA* long-acting β_2_-agonist, *LAMA* long-acting muscarinic antagonist, *SAMA* short-acting muscarinic antagonist, *PDE4* phosphodiesterase 4. *In the 12 months prior to indexing; therapy classes are not mutually exclusive. ICS, LABA, SAMA, SAMA/SABA, and PDE4 classes are not reported due to low patient numbers, ^†^Last therapy prior to indexing; regimens are mutually exclusive

Immediately prior to index (i.e., the last regimen prescribed before index), the most common maintenance treatments were MITT (prompt: 42.8%, delayed: 46.7%), ICS/LABA (prompt: 18.3%, delayed: 20.9%), and LABA/LAMA (prompt: 23.2%, delayed: 13.8%). Specific treatment regimens prescribed immediately prior to index are reported in Additional file 1: Table S2. The most common treatment regimens were FF/VI + UMEC (11.9%), UMEC + vilanterol trifenatate (9.7%), and fluticasone propionate/salmeterol xinafoate + tiotropium bromide (8.8%).

In the 12 months prior to the index date, patients in the prompt cohort had a mean of 11.0 all-cause and 2.7 COPD-related consultations; patients in the delayed cohort had a mean of 12.5 all-cause and 2.7 COPD-related consultations. Total costs were similar between cohorts in the 12 months prior to the index date (all-cause: £1600 for prompt vs. £1704 for delayed; COPD-related: £657 for prompt vs. £631 for delayed).

Baseline demographics, clinical characteristics, respiratory medication use, HCRU, and costs are also described for the additional analysis (when patients were categorized using the alternate definition of prompt initiation [i.e., initiation ≤ 14 days from index]) (Additional file 1: Tables S3 and S4).

### Rate of subsequent exacerbations following FF/UMEC/VI initiation

Results for the primary and secondary outcomes are presented for the weighted analyses (unless otherwise stated); the results from the unweighted analyses are included in the Supplementary appendix (Additional file 1: Tables S5–S10). Although absolute standardized mean differences (SMDs) of > 10% were observed for a number of variables in the unweighted data, the weighted data were adequately balanced (SMD < 10%) for most comparisons, except for “year of indexing 2017” where there were fewer patients in the prompt cohort when evaluating moderate/severe or moderate exacerbations. This imbalance was anticipated due to low patient numbers in 2017 as the index period start date was November 2017.

**Fig. 3:**
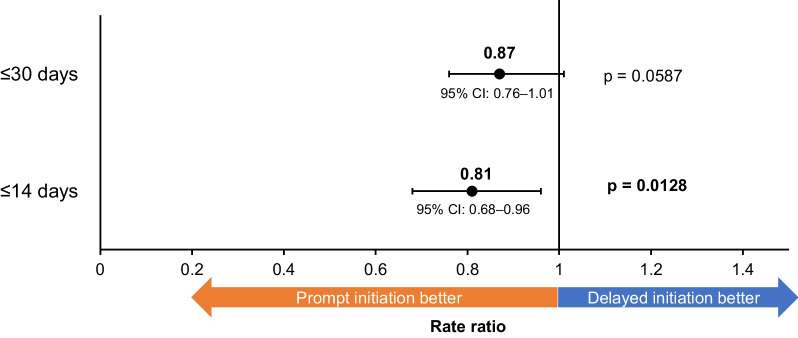
Rate of subsequent exacerbations following FF/UMEC/VI initiation. *FF/UMEC/VI* fluticasone furoate/umeclidinium/vilanterol, *CI* confidence interval. ≤ 14 days was performed as an additional analysis. p-values in bold text indicate statistical significance

Prompt initiators had numerically lower moderate/severe exacerbations compared with delayed initiators (incidence rate prompt: 0.0021, incidence rate delayed: 0.0024; RR: 0.87, 95% CI: 0.76–1.01, p = 0.0587; Fig. 3).

**Fig. 4:**
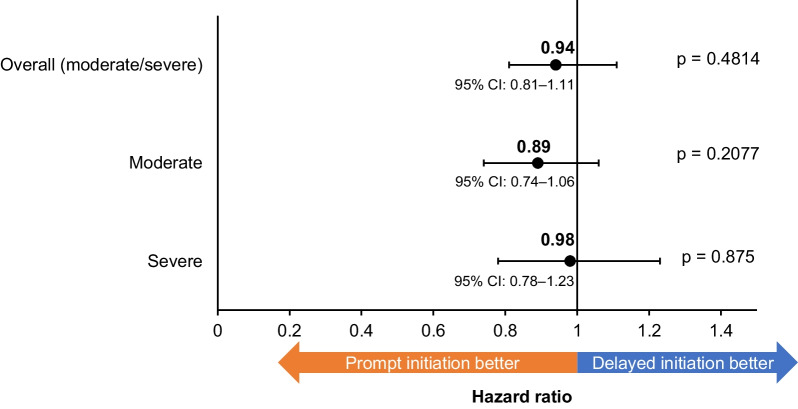
Time-to-first exacerbation following FF/UMEC/VI initiation. *FF/UMEC/VI* fluticasone furoate/umeclidinium/vilanterol, *CI* confidence interval

Similar results were observed when examining rates of moderate and severe exacerbations separately (moderate: incidence rate prompt: 0.0013, incidence rate delayed: 0.0015; RR: 0.87, 95% CI: 0.73–1.03, p = 0.1142; severe: incidence rate prompt: 0.0007, incidence rate delayed: 0.0008; RR: 0.87, 95% CI: 0.69–1.10, p = 0.2397). In the additional analysis, when ≤ 14 days was used as the cut-off point for prompt initiation, prompt initiators had significantly lower moderate/severe exacerbations compared with delayed initiators (RR: 0.81, 95% CI: 0.68–0.96, p = 0.0128; Fig. 3). Of note, the results from the unweighted analysis were very similar, suggesting limited potential for residual confounding (Additional file 1: Table S5). When patients were censored at the time of initiation of FF/UMEC/VI, the RR (95% CI) for prompt initiation was 0.69 (0.59–0.81, p < 0.0001). This is suggestive of a 31% reduction in risk of exacerbation due to direct benefits of FF/UMEC/VI itself vs. prior therapy, rather than the benefits of prompt initiation of FF/UMEC/VI. Time-to-first subsequent exacerbation did not differ significantly according to the timing of treatment initiation, though a slight trend towards longer median time-to-first moderate exacerbation could be observed among prompt vs. delayed initiators (Fig. 4).

A similar trend was observed when examining the rate of subsequent moderate/severe exacerbations, stratified by severity of index exacerbation (Additional file 1: Table S11). Prompt initiators had a numerically lower rate of combined (moderate and severe), moderate, and severe exacerbations compared with delayed initiators for both the moderate and severe index exacerbation weighted analyses, though these were not statistically significant.

### Hospital readmission following FF/UMEC/VI initiation

**Fig. 5:**
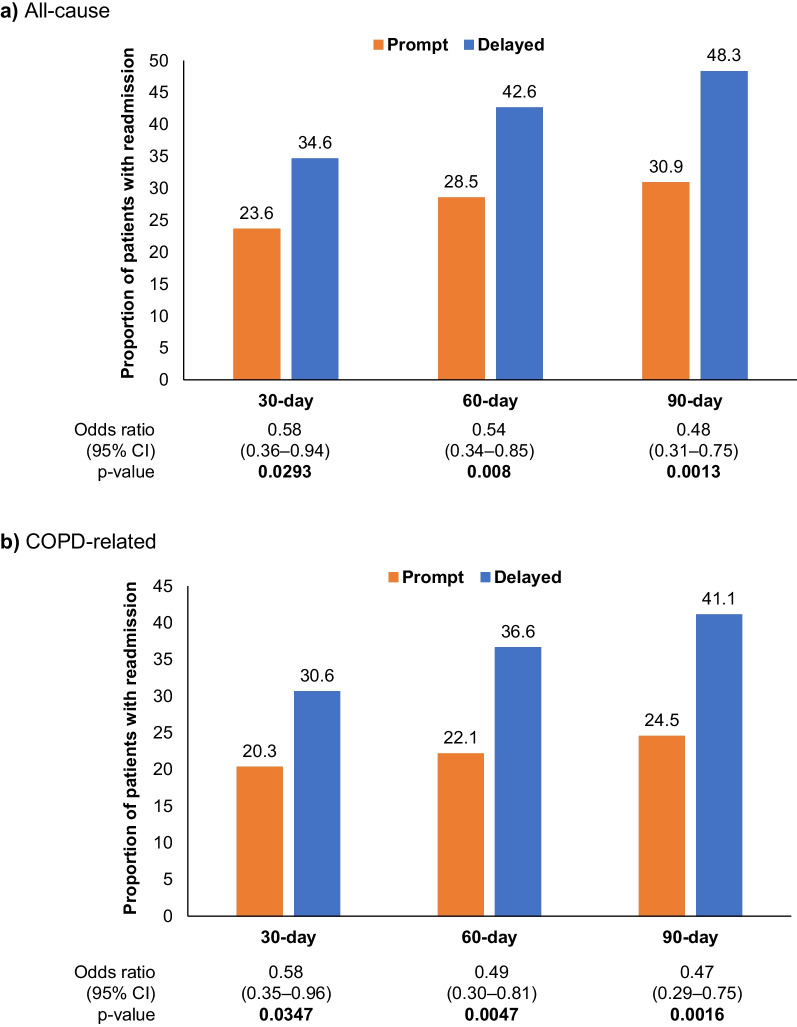
Hospital readmission following FF/UMEC/VI initiation. *FF/UMEC/VI* fluticasone furoate/umeclidinium/vilanterol, *CI* confidence interval, *COPD* chronic obstructive pulmonary disease. p-values in bold text indicate statistical significance

Both all-cause and COPD-related hospital readmissions were significantly lower among patients with prompt initiation compared with delayed initiation at the 30-, 60-, and 90-day time points (Fig. 5).

**Fig. 6:**
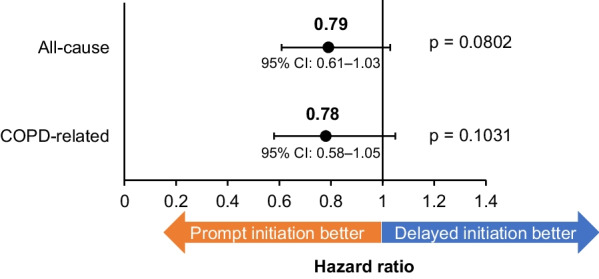
Time-to-first hospital readmission following initiation of FF/UMEC/VI. *FF/UMEC/VI* fluticasone furoate/umeclidinium/vilanterol, *CI* confidence interval, *COPD* chronic obstructive pulmonary disease

The proportion of patients with an all-cause 30-day readmission was 23.6% for prompt initiators and 34.6% for delayed initiators (odds ratio [95% CI]: 0.58 [0.36–0.95], p = 0.0293); the proportion of patients with a COPD-related 30-day readmission was 20.3% for prompt initiators and 30.6% for delayed initiators (odds ratio [95% CI]: 0.58 [0.35–0.96], p = 0.0347). Time-to-first all-cause and COPD-related hospital readmission were numerically longer among prompt initiators compared with delayed initiators (Fig. 6). All-cause readmission HR (95% CI) was 0.79 (0.61–1.03) and COPD-related readmission HR (95% CI) was 0.78 (0.58–1.05).

### HCRU and costs following FF/UMEC/VI initiation

**Fig. 7:**
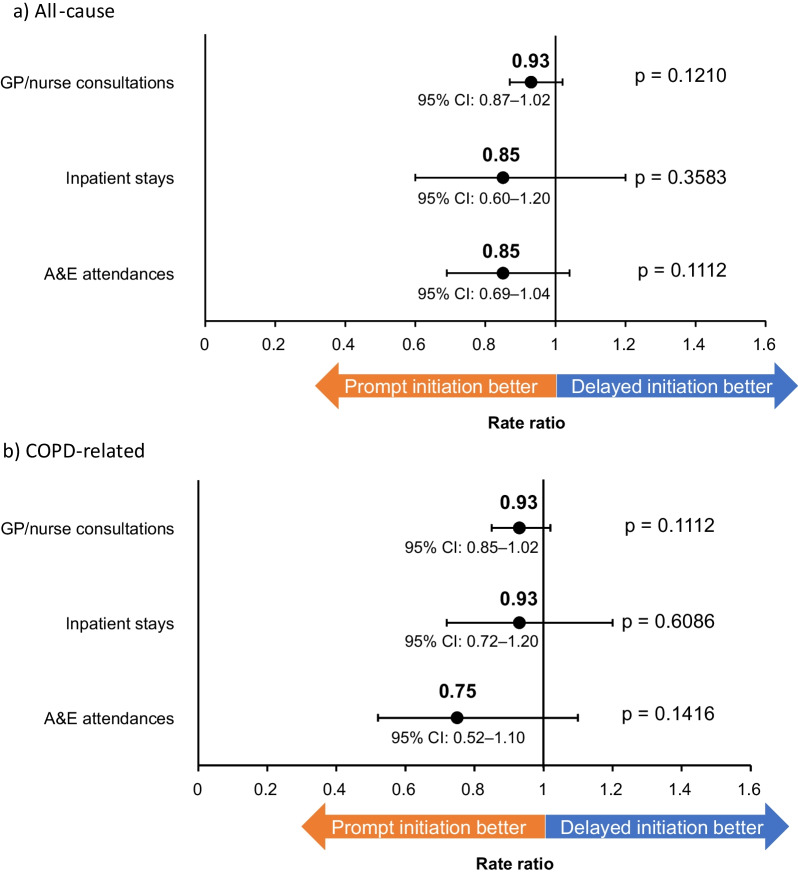
HCRU following FF/UMEC/VI initiation. *HCRU* healthcare resource utilization, *FF/UMEC/VI* fluticasone furoate/umeclidinium/vilanterol, *GP* general practitioner, *CI* confidence interval, *A&E* Accident and Emergency, *COPD* chronic obstructive pulmonary disease

All-cause and COPD-related HCRU rates per person-year were numerically lower among prompt initiators compared with delayed initiators (Fig. 7).

**Fig. 8:**
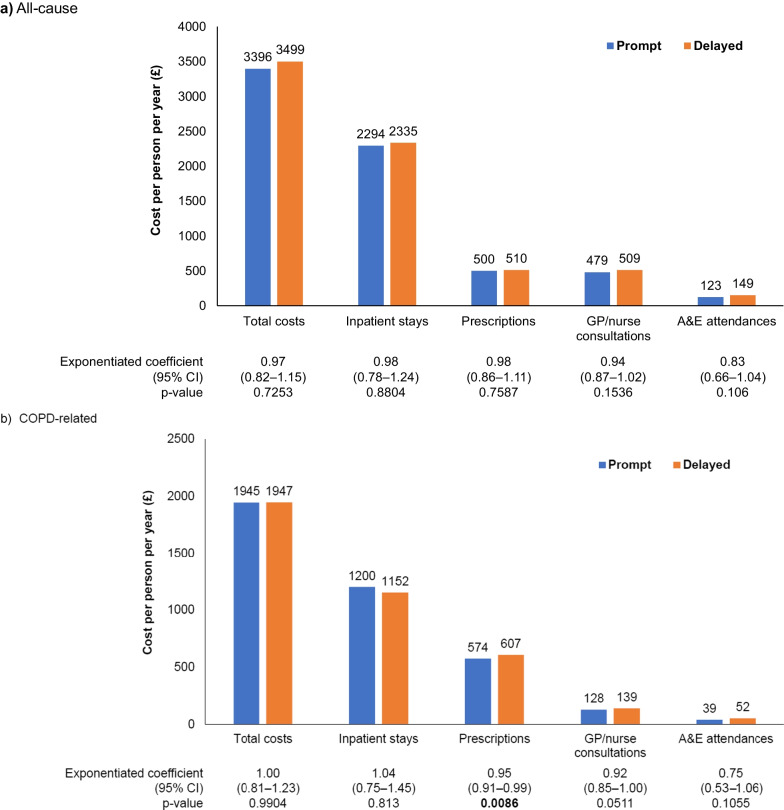
Total costs following FF/UMEC/VI initiation. *FF/UMEC/VI* fluticasone furoate/umeclidinium/vilanterol, *GP* general practitioner, *A&E* Accident and Emergency, *CI* confidence interval, *COPD* chronic obstructive pulmonary disease. p-values in bold text indicate statistical significance

Prompt initiators had numerically lower all-cause total costs and similar COPD-related costs per-person-per-year compared with delayed initiators (Fig. 8). COPD-related prescription costs were significantly lower among prompt initiators compared with delayed initiators (Fig. 8; prompt: £574, delayed: £607, p = 0.0086).

### Association between rate of subsequent exacerbations and time to FF/UMEC/VI initiation

When the association between rate of subsequent exacerbations and time-to-initiation of FF/UMEC/VI was examined as a continuous variable, significant associations were observed for moderate/severe exacerbations (RR per day of delayed initiation [95% CI]: 1.0018 [1.0005–1.0031], p = 0.0080) and moderate exacerbations (RR per day of delayed initiation [95% CI]: 1.0026 [1.0011–1.0041], p = 0.0010) (Table 3). Although the daily RRs were small, for moderate/severe exacerbations this would equate to an RR of 1.0127 per week of delayed initiation of FF/UMEC/VI.

**Table 3:** Association between rate of subsequent exacerbation and time-to-initiation of FF/UMEC/VI as a continuous metric

	RR	95% CI	p-value
Overall (moderate and severe) exacerbation	1.0018	(1.0005–1.0031)	**0.0080**
Moderate exacerbation	1.0026	(1.0011–1.0041)	**0.0010**
Severe exacerbation	1.0004	(0.9982–1.0027)	0.7010

*FF/UMEC/VI* fluticasone furoate/umeclidinium/vilanterol, *RR* rate ratio, *﻿CI* confidence interval. p-values in bold text indicate statistical significance

## Discussion

This study aimed to assess the outcomes of prompt vs. delayed initiation of SITT with FF/UMEC/VI among a cohort of patients with COPD in England. Initiation of FF/UMEC/VI within 0–30 days of an exacerbation was associated with numerically lower subsequent exacerbations compared with delayed initiation, though statistical significance was not reached. Low sample size, the ratio of prompt to delayed patients (1:3), or unknown/unmeasured variables not included in the model may have led to the study being underpowered, or otherwise impaired the ability to detect significant differences between the cohorts. When ≤ 14 days was used as the cut-off to define prompt initiation, prompt initiators had a significantly lower rate of subsequent exacerbations compared with delayed initiators. This implies that very prompt initiation may have a clinical benefit and that treatment should be started within 14 days of an exacerbation where possible. Of note, patients were not followed-up for the primary outcome until they initiated FF/UMEC/VI, preventing any time bias. In the main analysis, subsequent exacerbations were only assessed from the point of FF/UMEC/VI initiation (i.e., exacerbations occurring prior to initiation of FF/UMEC/VI were permitted but were not included in the outcome). The result of the additional analyses, censoring patients at initiation of FF/UMEC/VI, suggests that the benefit of prompt initiation is due to both efficacy of FF/UMEC/VI vs. prior therapy as well as benefit of prompt initiation of new therapy following an exacerbation.

Prompt initiation of FF/UMEC/VI following the index exacerbation was also associated with fewer all-cause and COPD-related hospital readmissions at all time points assessed, as well as lower COPD-related prescription costs compared with delayed initiation. A significant association was observed between time-to-treatment initiation (as a continuous variable) and rate of subsequent exacerbations. This indicates that timing of treatment has a bearing on clinical outcome and prognosis, suggesting that the rate of subsequent exacerbations may increase for each day of delayed initiation of FF/UMEC/VI following an exacerbation. This study included patients following the first exacerbation during the observation period (i.e., the first exacerbation in the previous 12 months). The findings suggest that physicians should consider a change of therapy after a single breakthrough exacerbation on prior maintenance therapy, rather than delaying change of therapy until after a patient has had several exacerbation events. The proportion of patients receiving each class of maintenance therapy immediately prior to index was similar among the prompt and the delayed cohorts for most regimens; however, there were more patients in the prompt cohort receiving LABA/LAMA immediately prior to index compared with the delayed cohort. Of note, around half of the included patients were using MITT immediately prior to their index exacerbation. Therefore, some patients receiving MITT remain uncontrolled and may benefit from an earlier switch to SITT.

The findings of this analysis are similar to previous studies. A retrospective study of over 10,000 patients with COPD in the US assessed the effects of prompt (≤ 30 days following index) vs. delayed (31–180 days following index) initiation of MITT following an exacerbation [7]. Total and severe exacerbation rates were 28.2% and 64.7% higher, respectively, in the delayed cohort compared with the prompt cohort (p < 0.0001). Total, medical, and prescription all-cause costs were 18.7%, 22.8%, and 8.8% higher, respectively, in the delayed cohort compared with the prompt cohort. Another retrospective study (using a similar design to the current study) of over 1000 patients with COPD in the US assessing the effect of prompt (≤ 30 days following index) vs. delayed (31–180 days following index) initiation of FF/UMEC/VI following an exacerbation reported that prompt patients had significantly lower rates of moderate/severe (RR [95% CI]: 0.79 [0.65–0.94], p = 0.004), moderate (RR [95% CI]: 0.84 [0.69–0.99], p = 0.038), and severe (RR [95% CI]: 0.57 [0.37–0.79], p = 0.002) exacerbations [9]. Mean all-cause and COPD-related total costs were also significantly lower among prompt initiators compared with delayed initiators.

Once-daily SITT with FF/UMEC/VI has previously been shown to be associated with a lower rate of moderate/severe exacerbations vs. dual therapy (FF/VI or UMEC/VI) [6] and significant improvements in lung function and health status vs. MITT [19]. FF/UMEC/VI has also been found to be a cost-effective treatment option compared with dual therapies and MITT [20–23]. The evidence from the current study may be useful in informing clinical guidance on the optimum management strategy for patients with COPD, particularly those hospitalized due to a severe exacerbation. It also highlights the lost potential for improved outcomes for the majority of patients in the study (75%) who were delayed initiators. Future studies may wish to prospectively investigate the effectiveness of prompt initiation of SITT upon discharge on reduction of future exacerbations and re-hospitalization. The data suggest that earlier initiation of FF/UMEC/VI could lessen the overall costs of intervention from healthcare professionals and pharmacological therapies used to manage the condition.

Although it is possible that similar findings may be observed for other SITTs, it should be noted that these data relate to FF/UMEC/VI only and may not be generalizable due to differences in constituent molecules, inhaler devices, and/or dosing frequency; therefore, caution should be taken when interpreting these findings in the context of other SITTs.

This study has a few potential limitations, which should be considered. Linkage to HES limits the sample to patients registered at a GP practice in England only; however, patient care/management is expected to be similar across the rest of the UK. Patients who died within 12 months of the index exacerbation have been excluded from the analysis, introducing the possibility of survivorship bias. Also, only medications prescribed in the primary care setting will have been captured; medications initiated in hospital and continued by the GP may have led to the incorrect classification of “delayed initiators” for some patients. However, this would be expected to result in a conservative estimate, thus reducing the observed effect of prompt initiation and biasing towards the null hypothesis.

## Conclusions

Compared with delayed initiation, prompt initiation of FF/UMEC/VI following a moderate/severe exacerbation was associated with fewer subsequent exacerbations, fewer hospital readmissions, and lower COPD-related prescription costs. These benefits lasted for at least 12 months.

### Supplementary Information

**Additional file 1.** Supplementary appendix.

## Data Availability

The datasets generated during and/or analyzed during the current study are available in the CPRD Aurum (https://www.cprd.com) and HES database (https://digital.nhs.uk/data-and-information/data-tools-and-services/data-services/hospital-episode-statistics). The data are provided by patients and collected by the NHS as part of their care and support. Authors had access to the study data for the purposes of this work only. The interpretation and conclusions contained in this study are those of the authors alone. Data were accessed through an existing GSK license to address the prespecified research questions only. Therefore, the data cannot be broadly disclosed or made publicly available at this time. Access to each database can be requested via the respective websites. Contains information from NHS Digital, licensed under the current version of the Open Government License. Copyright © (2022), re-used with the permission of The Health & Social Care Information Centre. All rights reserved.

## Abbreviations

COPD: Chronic obstructive pulmonary disease
FF: Fluticasone furoate
UMEC: Umeclidinium
VI: Vilanterol
CI: Confidence interval
ICS: Inhaled corticosteroid
LAMA: Long-acting muscarinic antagonist
LABA: Long-acting β2-agonist
MITT: Multiple-inhaler triple therapy
SITT: Single-inhaler triple therapy
CPRD: Clinical Practice Research Datalink
HES: Hospital Episode Statistics
A&E: Accident and Emergency
GP: General practitioner
IPTW: Inverse probability of treatment weighting
PS: Propensity score
BDP: Beclomethasone
FOR: Formoterol
GLY: Glycopyrronium bromide
RR: Rate ratio
HCRU: Healthcare resource utilization
KM: Kaplan–Meier
HR: Hazard ratio
SMD: Standardized mean difference
