# Correction: Benefit of prompt initiation of single-inhaler fluticasone furoate, umeclidinium, and vilanterol (FF/UMEC/VI) in patients with COPD in England following an exacerbation: a retrospective cohort study

**DOI:** 10.1186/s12931-024-02745-x

**Published:** 2024-04-01

**Authors:** Afisi S. Ismaila, Kieran J. Rothnie, Robert P. Wood, Victoria L. Banks, Lucinda J. Camidge, Alexandrosz Czira, Chris Compton, Raj Sharma, Shannon N. Millard, Olivia Massey, David M. G. Halpin

**Affiliations:** 1 Value Evidence and Outcomes, R&D Global Medical, GSK, 1250 South Collegeville Road, Collegeville, PA USA; 2 Department of Health Research Methods, Evidence and Impact, McMaster University, Hamilton, ON Canada; 3 Value Evidence and Outcomes, R&D Global Medical, GSK, London, UK; 4 Real-World Evidence, Adelphi Real World, Bollington, Cheshire UK; 5 Integrated Evidence Generation (Women’s Health Care), Bayer PLC, Reading, UK; 6 Global Medical, GSK, London, UK; 7 P1vital Limited, Wallingford, Oxfordshire UK; 8 College of Medicine and Health, University of Exeter Medical School, University of Exeter, Exeter, UK

**Correction: Respiratory Research (2023) 24: 229** 10.1186/s12931-023-02523-1

Following publication of the original article [1], the Authors identified errors in the COPD-related total costs for prompt and delayed initiators and the associated exponentiated coefficient (95% confidence interval) and p-value in Fig. 8b.

The corrected Fig. 8b is given below: 

These errors also impacted some statements under the “Results” and “Discussion” sections and “Conclusions”. This text has now been amended in this Correction.

## Results

*HCRU and costs following FF/UMEC/VI initiation.*

The text in the penultimate sentence under the heading “*HCRU and costs following FF/UMEC/VI initiation”* in the “Results” section originally read: Prompt initiators had numerically lower all-cause total costs and **significantly lower** COPD-related costs per-person-per-year compared with delayed initiators (Fig. 8; COPD-related costs: prompt £742, delayed £801, p = 0.0016).

Corrected sentence: Prompt initiators had numerically lower all-cause total costs and **similar** COPD-related costs per-person-per-year compared with delayed initiators (Fig. 8).

## Discussion

The first sentence of the second “Discussion” paragraph originally read: Prompt initiation of FF/UMEC/VI following the index exacerbation was also associated with fewer all-cause and COPD-related hospital readmissions at all time points assessed, **as well as lower COPD-related total costs and COPD-related prescription costs** compared with delayed initiation.

Corrected sentence: Prompt initiation of FF/UMEC/VI following the index exacerbation was also associated with fewer all-cause and COPD-related hospital readmissions at all time points assessed, **as well as lower COPD-related prescription costs** compared with delayed initiation.

## Conclusions

Finally, an additional discrepancy in the “Conclusions” section is noted.

The first sentence originally read: Compared with delayed initiation, prompt initiation of FF/UMEC/VI following a moderate/severe exacerbation was associated with fewer subsequent exacerbations, fewer hospital readmissions, and lower COPD-related **medical** costs.

Corrected sentence: Compared with delayed initiation, prompt initiation of FF/UMEC/VI following a moderate/severe exacerbation was associated with fewer subsequent exacerbations, fewer hospital readmissions, and lower COPD-related **prescription** costs.

The Authors apologise for these discrepancies and for any inconvenience to the journal and to the readers.

The original article has been corrected.
