# Supplementary material for: Benefit of prompt initiation of single-inhaler fluticasone furoate, umeclidinium, and vilanterol (FF/UMEC/VI) in patients with COPD in England following an exacerbation: a retrospective cohort study
Source: Respir Res. 2023 Sep 25;24:229. doi: 10.1186/s12931-023-02523-1 (PMC10521462; doi:10.1186/s12931-023-02523-1)
Supplement: Supplementary file 1 — Additional file 1. Supplementary appendix. [file 12931_2023_2523_MOESM1_ESM.docx]

**Additional file 1 Appendix**

**Additional file 1: methods**

**Identification of moderate and severe exacerbations from the Clinical Practice Research Datalink (CPRD)/Hospital Episode Statistics (HES)**

Exacerbations managed in primary care (i.e., recorded in CPRD) were considered moderate. Moderate exacerbations were identified from CPRD using a validated algorithm [1]. The algorithm was validated by case note review of 7136 events in 988 patients by two physicians and the resulting positive predictive value was >85% [1]. Moderate exacerbations were defined as the presence of a record for one of the four following events: prescriptions for both antibiotics and oral corticosteroids for a length of 5–14 days each (both prescriptions must have the same start date but each can last for a different number of days); two or more respiratory symptoms (breathlessness, cough, or sputum volume and/or purulence indicated by medical codes) and a prescription for antibiotics or oral corticosteroids (or both) on the same day; a lower respiratory tract infection medical code; or a chronic obstructive pulmonary disease exacerbation-specific medical code. Exacerbations resulting in hospitalization (i.e., recorded in HES) were considered severe. Severe exacerbations were identified using a validated algorithm, based on International Classification of Disease, 10^th^ Revision codes [2].

**Additional file 1: Table S1** Covariables included in the propensity score model

| **Variable category** | **Variable** |
| --- | --- |
| Demographics  *(at index date)* | 1. Age (in years) 2. Sex 3. Year 4. Geographic region 5. Socioeconomic status |
| Clinical characteristics  *(most recent measurement prior to and including the index date – unless otherwise stated)* | 1. Body mass index (kg/m^2^) 2. Forced expiratory volume in 1 s (%) 3. Medical Research Council score 4. Asthma diagnosis in 24 months prior to indexing (i.e., current) 5. Comorbidities prior to indexing (at any point):  - Depression - Anxiety - Gastroesophageal reflux disease - Acute myocardial infarction - Congestive heart failure - Stroke - Bronchiectasis - Dementia/cognitive impairment - Rheumatoid/osteo arthritis - Lung cancer - Diabetes  1. Smoking status prior to indexing |
| Medication use  *(during baseline)* | 1. Respiratory medications in 12 months prior to indexing:  - ICS - LABA - LAMA - ICS/LABA - ICS/SABA - LAMA/LABA - ICS/LAMA/LABA - SABA - SAMA - SABA/SAMA - Methylxanthines - PDE4 inhibitor (i.e., roflumilast)  1. Number of the above treatment classes/responses received in the 12 months prior to indexing (i.e., extent of polypharmacy) 2. Previous inhaled therapy (i.e., inhaled maintenance treatment strategy on the day prior to index)   ‐ MITT  ‐ ICS-LABA dual therapy  ‐ LABA-LAMA dual therapy  ‐ LAMA monotherapy  ‐ LABA monotherapy  ‐ ICS monotherapy  ‐ No maintenance treatment regimen |
| COPD-related HCRU  *(during baseline)* | 1. Number of primary care visits 2. Number of hospitalizations (exploratory objective 5 only) 3. Number of A&E visits |
| COPD-related medical costs  *(during baseline)* | 1. Total medical costs 2. Inpatient admission costs (exploratory objective 5 only) 3. Primary care costs 4. A&E costs |

*ICS*, inhaled corticosteroid, *LABA*, long-acting β_2_-agonist, *LAMA*, long-acting muscarinic antagonist, *SABA*, short-acting β_2_-agonist, *SAMA*, short-acting muscarinic antagonist, *PDE4*, phosphodiesterase 4, *MITT*, multiple-inhaler triple therapy, *COPD*, chronic obstructive pulmonary disease, *HCRU*, healthcare resource utilization, *A&E*, accident and emergency

**Additional file 1: Table S2** Specific drug combinations received immediately prior to index (i.e., last regimen before index; mutually exclusive)

|  | **Total  (N = 1599)** |
| --- | --- |
| Fluticasone furoate/vilanterol + umeclidinium bromide | 191 (11.9) |
| Umeclidinium bromide/vilanterol trifenatate | 155 (9.7) |
| Fluticasone propionate/salmeterol xinafoate + tiotropium bromide | 141 (8.8) |
| Tiotropium bromide | 94 (5.9) |
| Fluticasone furoate/vilanterol | 90 (5.6) |
| Budesonide/formoterol fumarate dihydrate + tiotropium bromide | 84 (5.3) |
| Fluticasone propionate/salmeterol xinafoate | 84 (5.3) |
| None | 78 (4.9) |
| Budesonide/formoterol fumarate dihydrate | 77 (4.8) |
| Beclometasone dipropionate/formoterol fumarate dihydrate + tiotropium bromide | 67 (4.2) |
| Beclometasone dipropionate/formoterol fumarate dihydrate | 64 (4.0) |
| Fluticasone furoate/vilanterol + tiotropium bromide | 52 (3.3) |
| Umeclidinium bromide | 49 (3.1) |
| Aclidinium bromide/formoterol fumarate dihydrate | 40 (2.5) |
| Glycopyrronium bromide/indacaterol maleate | 28 (1.8) |
| Beclometasone dipropionate/formoterol fumarate dihydrate + umeclidinium bromide | 27 (1.7) |
| Fluticasone propionate/salmeterol xinafoate + umeclidinium bromide | 24 (1.5) |
| Olodaterol hydrochloride/tiotropium bromide | 19 (1.2) |
| Beclometasone dipropionate | 18 (1.1) |
| Aclidinium bromide + budesonide/formoterol fumarate dihydrate | 16 (1.0) |
| Aclidinium bromide | 15 (0.9) |
| Aclidinium bromide + fluticasone propionate/salmeterol xinafoate | 15 (0.9) |
| Glycopyrronium bromide | 14 (0.9) |
| Budesonide/formoterol fumarate dihydrate + glycopyrronium bromide | 12 (0.8) |
| Aclidinium bromide + beclometasone dipropionate/formoterol fumarate dihydrate | 11 (0.7) |
| Beclometasone dipropionate/formoterol fumarate dihydrate + glycopyrronium bromide | 11 (0.7) |
| Other | 123 (7.7) |

**Additional file 1: Table S3** Baseline demographics of patients stratified by prompt (≤14 days) or delayed initiation of FF/UMEC/VI

|  | **Prompt  (0–14 days)**  **(N = 223)** | **Delayed (15–180 days)**  **(N = 1376)** |
| --- | --- | --- |
| Age at index (years) |  |  |
| Mean (SD) | 69.3 (10.1) | 69.9 (10.4) |
| Sex, n (%) |  |  |
| Male | 126 (56.5) | 721 (52.4) |
| Ethnicity, n (%) |  |  |
| White | 213 (95.5) | 1307 (95.0) |
| Smoking status, n (%) |  |  |
| Current smoker | 120 (53.8) | 691 (50.2) |
| Former smoker | 103 (46.2) | 685 (49.8) |
| BMI (kg/m^2^) | n = 201 | n = 1228 |
| Mean (SD) | 27.0 (6.3) | 27.4 (6.3) |
| FEV_1_/FVC ratio | n = 138 | n = 835 |
| Mean (SD) | 55.7 (14.0) | 56.4 (14.6) |
| FEV_1_% predicted | n = 187 | n = 1102 |
| Mean (SD) | 54.8 (18.5) | 55.8 (19.7) |
| MRC Dyspnea Scale score, n (%) |  |  |
| Grade 1 | 17 (7.6) | 97 (7.1) |
| Grade 2 | 64 (28.7) | 383 (27.8) |
| Grade 3 | 67 (30.0) | 424 (30.8) |
| Grade 4 | 52 (23.3) | 270 (19.6) |
| Grade 5 | 7 (3.1) | 59 (4.3) |
| Unknown | 16 (7.2) | 143 (10.4) |
| Comorbidities, n (%) |  |  |
| Depression | 113 (50.7) | 625 (45.4) |
| Rheumatoid/osteo arthritis | 80 (35.9) | 532 (38.7) |
| Anxiety | 66 (29.6) | 439 (31.9) |
| Gastroesophageal reflux disease | 59 (26.5) | 391 (28.4) |
| Diabetes | 50 (22.4) | 309 (22.5) |
| Congestive heart failure | 36 (16.1) | 136 (9.9) |
| Stroke | 27 (12.1) | 167 (12.1) |
| Acute myocardial infarction | 26 (11.7) | 160 (11.6) |
| Dementia/cognitive impairment | 24 (10.8) | 146 (10.6) |
| Bronchiectasis | 15 (6.7) | 98 (7.1) |
| Lung cancer | < 5 | 21 (1.5) |

*FF/UMEC/VI* fluticasone furoate/umeclidinium/vilanterol, *SD* standard deviation, *BMI* body mass index, *FEV_1_* forced expiratory volume in 1 s, *FVC* forced vital capacity, *MRC* Medical Research Council

**Additional file 1: Table S4** Treatment patterns at baseline for patients stratified by prompt (≤14 days) or delayed FF/UMEC/VI initiation

|  | **Prompt (0–14 days)**  **(N = 223)** | **Delayed (15–180 days)**  **(N = 1376)** |
| --- | --- | --- |
| Number of respiratory therapy classes at baseline  Mean (SD) |  |  |
|  | 3.15 (1.1) | 2.95 (1.2) |
| Class of respiratory therapy at baseline*, n (%) |  |  |
| SABA | 207 (92.8) | 1219 (88.6) |
| MITT | 152 (68.2) | 853 (62.0) |
| ICS/LABA | 140 (62.8) | 812 (59.0) |
| LAMA | 113 (50.7) | 701 (50.9) |
| LABA/LAMA | 57 (25.6) | 256 (18.6) |
| Methylxanthine | 16 (7.2) | 82 (6.0) |
| ICS/SABA | 7 (3.1) | 66 (4.8) |
| Inhaled therapy regimen immediately prior to index^†^, n (%) |  |  |
| MITT | 98 (44.0) | 633 (46.0) |
| LABA/LAMA | 50 (22.4) | 207 (15.0) |
| ICS/LABA | 44 (19.7) | 280 (20.4) |
| Other^‡^ | 31 (13.9) | 256 (18.6) |

*FF/UMEC/VI* fluticasone furoate/umeclidinium/vilanterol, *SD* standard deviation, *SABA* short-acting β_2_‑agonist, *MITT* multiple-inhaler triple therapy, *ICS* inhaled corticosteroid, *LABA* long-acting β_2_-agonist, *LAMA* long-acting muscarinic antagonist, *SAMA* short-acting muscarinic antagonist,
*PDE4* phosphodiesterase 4. *In the 12 months prior to indexing. Therapy classes are not mutually exclusive. ICS, LABA, SAMA, SAMA/SABA, and PDE4 classes are not reported due to low patient numbers, ^†^Last therapy prior to indexing; regimens are mutually exclusive, ^‡^Includes patients receiving ICS, LABA, LAMA, and none of the above maintenance treatment

**Additional file 1: Table S5** Rate of subsequent exacerbations following FF/UMEC/VI initiation – unweighted analysis

|  | **RR** | **95% CI** | **p-value** |
| --- | --- | --- | --- |
| Overall (moderate/severe) | 0.90 | 0.79–1.03 | 0.1373 |
| Moderate | 0.84 | 0.71–0.99 | **0.0372** |
| Severe | 1.04 | 0.82–1.30 | 0.7662 |

*FF/UMEC/VI* fluticasone furoate/umeclidinium/vilanterol, *RR* rate ratio, *CI* confidence interval. p-values in bold text indicate statistical significance

**Additional file 1: Table S6** Time to first exacerbation following FF/UMEC/VI initiation – unweighted analysis

|  | **HR** | **95% CI** | **p-value** |
| --- | --- | --- | --- |
| Overall (moderate/severe) | 0.95 | 0.82–1.11 | 0.5320 |
| Moderate | 0.86* | 0.73–1.03 | 0.0959 |
| Severe | 1.09^†^ | 0.87–1.36 | 0.4518 |

*FF/UMEC/VI* fluticasone furoate/umeclidinium/vilanterol, *HR* hazard ratio, *CI* confidence interval. *Proportional hazards assumption violated (p = 0.0334), ^†^proportional hazards assumption violated (p = 0.0264)

**Additional file 1: Table S7** Hospital readmission following FF/UMEC/VI initiation – unweighted analysis

|  | **OR (prompt:delayed)** | **95% CI** | **p-value** |
| --- | --- | --- | --- |
| All-cause |  |  |  |
| 30-day | 0.53 | 0.33–0.83 | **0.0057** |
| 60-day | 0.49 | 0.32–0.75 | **0.0011** |
| 90-day | 0.45 | 0.30–0.69 | **0.0002** |
| COPD-related |  |  |  |
| 30-day | 0.52 | 0.32–0.84 | **0.0070** |
| 60-day | 0.45 | 0.29–0.72 | **0.0008** |
| 90-day | 0.43 | 0.27–0.67 | **0.0002** |

*FF/UMEC/VI* fluticasone furoate/umeclidinium/vilanterol, *OR*, odds ratio, *CI* confidence interval,
*COPD* chronic obstructive pulmonary disease. p-values in bold text indicate statistical significance

**Additional file 1: Table S8** Time to first hospital readmission following FF/UMEC/VI initiation – unweighted analysis

|  | **HR** | **95% CI** | **p-value** |
| --- | --- | --- | --- |
| All-cause | 0.75 | 0.59–0.96 | **0.0237** |
| COPD-related | 0.74 | 0.56–0.97 | **0.0312** |

*FF/UMEC/VI* fluticasone furoate/umeclidinium/vilanterol, *HR* hazard ratio, *CI* confidence interval,
*COPD* chronic obstructive pulmonary disease. p-values in bold text indicate statistical significance

**Additional file 1: Table S9** HCRU following FF/UMEC/VI initiation – unweighted analysis

|  | **RR** | **95% CI** | **p-value** |
| --- | --- | --- | --- |
| All-cause |  |  |  |
| GP/nurse consultations | 0.91 | 0.84–0.98 | **0.0107** |
| Inpatient stays | 0.91 | 0.64–1.29 | 0.5913 |
| A&E attendances | 0.91 | 0.76–1.10 | 0.3222 |
| COPD-related |  |  |  |
| GP/nurse consultations | 0.90 | 0.83–0.98 | **0.0108** |
| Inpatient stays | 1.13 | 0.87–1.46 | 0.3745 |
| A&E attendances | 1.00 | 0.70–1.44 | 0.9879 |

*HCRU* healthcare resource utilization, *FF/UMEC/VI* fluticasone furoate/umeclidinium/vilanterol, *RR* rate ratio, *CI* confidence interval, *GP* general practitioner, *A&E* accident and emergency, *COPD* chronic obstructive pulmonary disease. p-values in bold text indicate statistical significance

**Additional file 1: Table S10** Total costs following FF/UMEC/VI initiation – unweighted analysis

|  | **Exponentiated coefficient** | **95% CI** | **p-value** |
| --- | --- | --- | --- |
| All-cause |  |  |  |
| Total | 1.00 | 0.86–1.17 | 0.9720 |
| Inpatient stays | 1.03 | 0.83–1.28 | 0.7583 |
| Prescriptions | 0.97 | 0.85–1.10 | 0.6316 |
| GP/nurse consultations | 0.91 | 0.84–0.98 | **0.0132** |
| A&E attendances | 0.94 | 0.76–1.15 | 0.5301 |
| COPD-related |  |  |  |
| Total | 0.95 | 0.91–0.99 | **0.0123** |
| Inpatient stays | 1.11 | 0.84–1.47 | 0.4779 |
| Prescriptions | 0.96 | 0.92–0.99 | **0.0166** |
| GP/nurse consultations | 0.90 | 0.83–0.97 | **0.0071** |
| A&E attendances | 0.99 | 0.68–1.45 | 0.9724 |

*FF/UMEC/VI* fluticasone furoate/umeclidinium/vilanterol, *CI* confidence interval, *GP* general practitioner, *A&E* accident and emergency, *COPD* chronic obstructive pulmonary disease. p-values in bold text indicate statistical significance

**Additional file 1: Table S11** Rate of subsequent exacerbations following FF/UMEC/VI initiation, stratified by severity of index exacerbation

|  | **Weighted** | | **Unweighted** | |
| --- | --- | --- | --- | --- |
|  | **RR (95% CI)** | **p-value** | **RR (95% CI)** | **p-value** |
| Subsequent overall exacerbations (moderate and severe) | | | | |
| Moderate index exacerbation | 0.9220 (0.7682–1.1067) | 0.3835 | 0.9021 (0.7598–1.0712) | 0.24 |
| Severe index exacerbation | 0.8157 (0.6478–1.0271) | 0.0831 | 0.8656 (0.6957–1.0769) | 0.1951 |
| Subsequent moderate exacerbations | | | | |
| Moderate index exacerbation | 0.900 (0.7307–1.1107) | 0.3286 | 0.8516 (0.6962–1.0418) | 0.1183 |
| Severe index exacerbation | 0.9176 (0.6453–1.3048) | 0.632 | 0.9098 (0.6709–1.2337) | 0.5427 |
| Subsequent severe exacerbations | | | | |
| Moderate index exacerbation | 0.9785 (0.6884–1.3906) | 0.9033 | 1.0293 (0.7387–1.4345) | 0.8643 |
| Severe index exacerbation | 0.7520 (0.5363–1.0545) | 0.0985 | 0.8273 (0.6115 – 1.1194) | 0.2193 |

*FF/UMEC/VI* fluticasone furoate/umeclidinium/vilanterol, *RR* rate ratio, *CI* confidence interval

**Additional file 1: Fig. S1** Distribution of time-to-initiation of FF/UMEC/VI


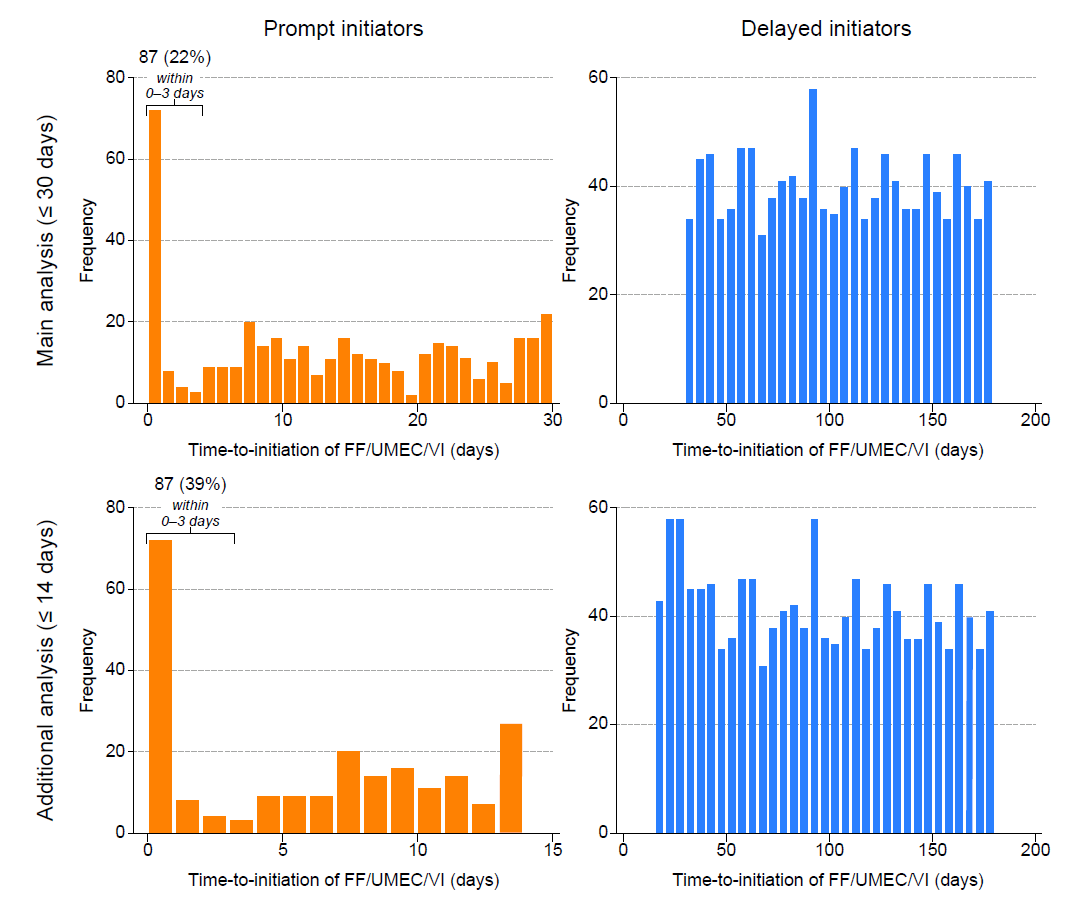


*FF/UMEC/VI* fluticasone furoate/umeclidiniumm/vilanterol

**Additional file 1: references**

1. Rothnie KJ, Müllerová H, Hurst JR, et al. Validation of the recording of acute exacerbations of COPD in UK primary care electronic healthcare records. PLoS One. 2016;11(3):e0151357.

2. Rothnie KJ, Müllerová H, Thomas SL, et al. Recording of hospitalizations for acute exacerbations of COPD in UK electronic health care records. Clin Epidemiol. 2016;8:771-82.
